# Rapid Inflammasome Activation Is Attenuated in Post-Myocardial Infarction Monocytes

**DOI:** 10.3389/fimmu.2022.857455

**Published:** 2022-04-26

**Authors:** Hector Giral, Vedran Franke, Minoo Moobed, Maja F. Müller, Laura Lübking, Divya Maria James, Johannes Hartung, Kira Kuschnerus, Denitsa Meteva, Claudio Seppelt, Philipp Jakob, Roland Klingenberg, Nicolle Kränkel, David Leistner, Tanja Zeller, Stefan Blankenberg, Friederike Zimmermann, Arash Haghikia, Thomas F. Lüscher, Altuna Akalin, Ulf Landmesser, Adelheid Kratzer

**Affiliations:** ^1^ Department of Cardiology, Charité - Universitätsmedizin Berlin, Corporate Member of Freie Universität Berlin, Humboldt-Universität zu Berlin, Berlin, Germany; ^2^ DZHK (German Centre for Cardiovascular Research), Partner Site Berlin, Berlin, Germany; ^3^ Max Delbrück Center, The Berlin Institute for Medical Systems Biology, Berlin, Germany; ^4^ Department of Cardiology, University Hospital Zurich, Zurich, Switzerland; ^5^ Berlin Institute of Health at Charité – Universitätsmedizin Berlin, Berlin, Germany; ^6^ Department of Cardiology, University Heart and Vascular Center, Hamburg, Germany; ^7^ DZHK (German Center for Cardiovascular Research), Partner Site Hamburg, Lübeck, Kiel, Hamburg, Germany; ^8^ Center for Molecular Cardiology, University of Zurich, Zurich, Switzerland

**Keywords:** inflammasome, interleukin 18, monocytes, acute myocardial infarction, refractory behavior, IRAKM, TNFAIP3 (A20)

## Abstract

Inflammasomes are crucial gatekeepers of the immune response, but their maladaptive activation associates with inflammatory pathologies. Besides canonical activation, monocytes can trigger non-transcriptional or rapid inflammasome activation that has not been well defined in the context of acute myocardial infarction (AMI). Rapid transcription-independent inflammasome activation induced by simultaneous TLR priming and triggering stimulus was measured by caspase-1 (CASP1) activity and interleukin release. Both classical and intermediate monocytes from healthy donors exhibited robust CASP1 activation, but only classical monocytes produced high mature interleukin-18 (IL18) release. We also recruited a limited number of coronary artery disease (CAD, n=31) and AMI (n=29) patients to evaluate their inflammasome function and expression profiles. Surprisingly, monocyte subpopulations isolated from blood collected during percutaneous coronary intervention (PCI) from AMI patients presented diminished CASP1 activity and abrogated IL18 release despite increased NLRP3 gene expression. This unexpected attenuated rapid inflammasome activation was accompanied by a significant increase of TNFAIP3 and IRAKM expression. Moreover, TNFAIP3 protein levels of circulating monocytes showed positive correlation with high sensitive troponin T (hsTnT), implying an association between TNFAIP3 upregulation and the severity of tissue injury. We suggest this monocyte attenuation to be a protective phenotype aftermath following a very early inflammatory wave in the ischemic area. Damage-associated molecular patterns (DAMPs) or other signals trigger a transitory negative feedback loop within newly recruited circulating monocytes as a mechanism to reduce post-injury tissue damage.

## Introduction

Monocytes orchestrate both onset and resolution of inflammation as vital players in the control of pathogen-mediated infections and sterile injuries (1). Numerous studies have associated the inflammatory burden due to exacerbated monocyte response with increased severity and worse outcome of multiple diseases, including acute myocardial infarction (AMI) (2).

Inherent plasticity of human circulating Mo results in phenotypic diversity leading to a classification into three major subpopulations: classical (CD14^++^CD16^-^), intermediate (CD14^++^CD16^+^), and non-classical monocytes (CD14^+^CD16^++^) (3). In turn, mice studies have used Ly6C as a marker to define classical (Ly6Chigh) and non-classical (Ly6Clow) subpopulations although in cell fate mapping studies these subpopulations are also defined by high expression of CCR2 or CX3CR1, respectively (4). Different functional roles have been described for monocyte subpopulations in AMI (5). Non-classical monocytes have the ability to patrol not only the luminal venous vascular endothelium (6) but also the arterial vasculature (7), a behavior promoting endothelial repair and regeneration (8). Increased numbers if human circulating CD16+ Mo (INT) have been previously associated with worse outcome of cardiovascular disease emerging as a potential clinical marker (9). At the time of an ischemic event, such as in AMI, monocytes are mobilized from spleen and bone marrow potentially undertaking different spatiotemporal functions (10). While in the early phase after an ischemic incident mainly classical monocytes are recruited to the infarcted core, at the second, post-infarct phase equal amounts of recruited CD14^++^CD16^-^ and CD14^++^CD16^+^ cells have been observed undertaking diverse functions (11).

Inflammasomes comprise a group of genes acting as intracellular sensors that form multiprotein complexes and are considered key players of the inflammatory response as important mediators of monocyte functionality (12). They respond to specific inflammatory cues by triggering the multimerization of a complex constituted by NOD-like receptors (NLRs), the adaptor protein PYD and CARD domain containing (PYCARD) and caspase-1 (CASP1). Formation of this complex induces catalytic activation of CASP1 that subsequently promotes proteolytic processing of pro-inflammatory interleukin 1 beta (IL-1β) and interleukin 18 (IL18) (13). Despite the essential role of inflammasome complexes in immune surveillance, increasing evidence suggests that inflammasome dysregulation drives the course of chronic inflammatory diseases (14). Nonetheless, inflammasome activation can also undergo suppression as observed in severe trauma (15). Such traumatic experiences can be also faced to various degrees by cardiac tissue areas upon an ischemic insult. The impact of inflammasome function on atherosclerosis and myocardial infarction has been established in various studies with special emphasis on NLRP3 (16) and its transcriptional activation. Additionally, genetic variants of NLRC4 inflammasome were associated to cardiovascular disease (17). NLRC4 activation by nucleotide-derived metabolites has been associated to chronic inflammation in a certain group of elderly people with constitutive expression of IL-1β resulting in low-grade interleukin release (18).

Inflammasome activation is tightly regulated by an increasing number of modulators whose precise underlying mechanisms of action, although being steadily unfolded, are not fully resolved. Canonical activation of NLRP3, the most extensively examined inflammasome, demands a two-signal activation. First a priming step that increases NLRP3 expression and next a second stimulus, which triggers the assembly of the multiprotein complex. Within this classical two-signal model, inflammasome activation fully depends on *de novo* transcriptional production of inflammasome components such as NLRP3 and pro-IL-1β (19). In contrast, an alternative transcription-independent activation can be triggered, particularly in monocytes. Under this condition, simultaneous priming with Toll-like receptors 2 or 4 (TLR2 or TLR4) and ATP-mediated stimulation is sufficient to trigger non-transcriptional or rapid inflammasome activation. Previous studies have reported that rapid inflammasome activation solely relies on constitutive inflammasome components regulated by post-translational modifications, such as ubiquitination or phosphorylation (20–22), extensively reviewed elsewhere (23). A recent study highlighted priming independent inflammasome activation and mature IL18 release upon nigericin treatment compared to 4h LPS pre-stimulation in both healthy CD14^+^ monocytes and THP1 cells (24).

Transcription-dependent inflammasome activation and resulting interleukin release has been intensively studied in cardiovascular disease. In certain atherosclerosis models NLRP3 blockage reduced plaque size (25) and myocardial injury after ischemia reperfusion (26). In secondary prevention of cardiovascular events in patients with previous acute myocardial infarction (AMI), blockage of IL-1β performed within the CANTOS trial suggested the potential of NLRP3 as a target for AMI therapies (27). NLRP3 inhibition at different cardiovascular disease stages can potentially help in prevention or improvement of heart failure development.

We consider that the acute inflammasome activation and regulation may be concomitant to severe tissue injury where cell damage leads to massive release of intracellular Damage-associated molecular patterns (DAMPs) and ATP. With inflammasome components such as NLRP3 highly expressed and readily available in monocytes together with constitutively expressed pro-IL18 anytime accessible for cleavage the inflammasome complex demands strict and tight control. Different post-transcriptional and post-translational mechanisms provide a control for the fast-acting machinery available to keep a balance between pro-and anti-inflammatory phases. One of these molecules is the ubiquitin-editing enzyme TNFAIP3, also known as A20, which is shown to preserve immune homeostasis by inhibiting the Myd88 and TRIF dependent TLR signaling (28).

However, the underlying mechanisms of rapid inflammasome activation have not been fully characterized and no previous reports have explored its role in human monocyte subpopulations in the course of AMI. In previous studies (29) tolerogenic behavior has been associated to mitochondrial DNA acting as DAMPs in AMI patients and leading to transcription-dependent upregulation of IRAKM, an inactive kinase and negative regulator of TLR signaling in monocyte-derived macrophages. In contrast to numerous previous studies focusing on transcription-dependent inflammasome activation and regulation, our present study aimed to unravel for the first time a functional analysis of rapid inflammasome activation in human monocyte subpopulations. Furthermore, we analyzed monocyte subpopulations obtained from AMI and stable coronary artery disease (CAD) patients providing novel insights into the behavior of these plastic immune cells in the frame of an acute inflammatory incident.

## Methods

### Patient and Healthy Subject Characteristics and Isolation

After receiving the consent from each participant, 40-80mL arterial blood from AMI patients were drawn within 24 to 72h of the first pain onset during a PCI. According to our ethically approved inclusion criteria, patients did not have any chronic or acute inflammatory diseases. Age-matched CAD patients were chosen according to the definition of an occlusion of at least 50% in one main coronary artery. Healthy donors in the same age range did not take any medication, had no family history of AMI, a BMI<30 and no defined cardiovascular risk factors as well as no chronic or acute diseases at the time of blood draw. Blood analysis and an echocardiogram were performed in healthy donors in order to qualify for the inclusion in the study. General clinical parameters can be found in **Supplementary Table 1**. Blood was collected in 10mL BD Vacutainer blood collection tubes with K2 EDTA and cells were isolated from blood within the first hour of collection. All subjects read the patient information and gave their written consent, and the study has been conducted according to the declaration of Helsinki and was approved by the ethics committee in Zurich [Kantonale Ethik-Kommission Zürich (KEK)], Switzerland (KEK-ZH-Nr. 2012-0321) and the Berlin State Ethics Committee in Berlin, Germany (EA4/122/14 and EA1/270/16).

### Isolation of Monocyte Subpopulations

Fresh blood was two-fold diluted with pyrogen-free PBS, overlaid onto the Ficoll-Paque (d=1.077) density solution (GE Healthcare), centrifuged for 30 min at 500g with reduced acceleration and no break at room temperature (RT) and then peripheral blood mononuclear cells (PBMC) were collected from the interphase. Contaminant platelets were removed by two consecutive centrifugation steps at 100g for 15min at 4°C. PBMC underwent magnetic cell sorting (MACS) using CD14-labeled magnetic beads (Miltenyi Biotec) and using MACS buffer (pyrogen-free PBS, 0.5% BSA, 2mM EDTA) to obtain CD14^+^ monocytes. These total CD14^+^ monocytes were further separated into classical, intermediate and non-classical monocyte subpopulations by labelling them with CD14-APC (Biolegend) and CD16-PercpCy5.5 (Biolegend) FACS antibodies for 30min at 4°C in the dark (see gating and sorting strategy in **Figure S1A**). Unbound antibodies were washed away with MACS buffer by centrifugation at 4°C and clumping was avoided by filtering the cell suspension through a 70μm filter tube. Cells were then collected on a BD FACS Aria II cell sorter using a 70μm nozzle according to best practice and defined minimal standards (30). Cells were collected in 5mL round-bottom polypropylene FACS tubes containing 1mL ice-cold RPMI (2g/L glucose) with 2% human AB serum (Sigma), HEPES, Na-Pyruvate, glutamine and penicilin/streptomycin. Freshly sorted cells were right away split into different fractions: either lysed in Qiazol (Qiagen) for RNA analysis, or in RIPA buffer plus protease inhibitors for protein analysis or kept in RPMI media for functional assays such as ELISA, Caspase-1(CASP1) activity FLICA assay and LDH measurements.

### RNA Isolation and Real Time PCR

RNA was isolated from classical, intermediate or non-classical monocyte subpopulations or CD14+ monocytes using total RNA isolation kits Qiagen miRNeasy Mini or miRNeasy Micro (Qiagen). RNA quality (A260/A280 ratio) and concentration was determined on a NanoVue Spectrophotometer (VWR) and reverse-transcription was performed using a High-Capacity cDNA Reverse Transcription Kit (Applied Biosystems). Real-time PCR was performed on a Viia7 real time PCR instrument (ThermoFisher) using the 384-well block insert. We used both SYBR green and Taqman assays (list of primers provided in **Supplementary Table 2**) and the respective Master mixes. After 40 amplification cycles a melting curve analysis was performed for SYBR green primers. Expression levels were calculated using the ΔΔCt method normalised to the housekeeping gene RPL28. Primer sequences for SYBR green PCR analysis were used from PrimerBank and if not available designed *via* NCBI Primer-BLAST. All primers for SYBR green analysis were synthesized by TIB MOLBIOL and upon arrival dissolved in nuclease-free water and stored in stock solutions of 100μM. For all SYBR primers efficiencies were calculated using a standard curve and only efficiencies between 90% and 110% were considered appropriate; all the primers were used at a final concentration of 0.2μM. Assay numbers for Taqman probes are denoted in the **Supplementary Table 2**.

### Caspase-1 Activity Assay

Freshly isolated and FACS sorted classical, intermediate or non-classical monocytes were plated on 384-well plates (25000 cells/well). Cells were pre-treated with or without MCC950 (10µM) under shaking at 37°C for 15min prior to simultaneous treatment with ATP (2mM) and LPS (500ng/mL) for 45 min. Concurrently, FAM-labeled YVAD-cmk peptide (FAM-FLICA Caspase-1 Assay, ImmunoChemistry Technologies) was added during the last 30min of treatment. After treatment cells were washed twice using an apoptosis wash buffer (provided in the assay kit) and fluorescence signal was measured on a FACS Attune II flow cytometer with excitation of the blue laser at 488nm and detection in the BL1 channel (FITC) using an emission filter of 530/30. Flow cytometer data was acquired at a flow rate of 100μL/min and a total volume of 100μL. Our gating strategy comprised a singlets gate applying forward scatter height versus forward scatter area in order to avoid false positive signals from doublets. The same threshold cut-off for dead cells was used and positivity of the FAM-FLICA signal was detected in the BL1 channel of Attune II as FAM/FLICA^high/low^. Measurement results are shown as arbitrary fluorescence units of median fluorescence intensity.

Analysis was performed using the Kaluza software from Beckman Coulter and measurements were calculated as Median Fluorescence Intensity (MFI) in arbitrary fluorescence units.

### Western Blot Analysis

Freshly isolated monocyte subpopulations treated as described in previous section were lysed in RIPA lysis buffer containing full cocktails of protease and phosphatase inhibitors. Protein concentration was measured using the BCA assay and western blot samples were prepared at a concentration of 0.2µg/uL using Laemmli buffer (LB) and heated at 95°C for 5min. Polyacrylamide gels (12%) were loaded with 1 µg total protein per sample and run at 50V for 20min and 100V for 2h. Wet-transfer to ultrahydrophobic 0.22 µm PVDF membranes (Millipore) was performed for 2h at constant current of 200mA using constant stirring and an icepack for cooling. Membranes were blocked for 30min with 5% non-fat milk and incubated overnight with respective primary antibodies (see **Supplementary Table 2**) in PBST (PBS, 0.5% Tween-20) containing 1% BSA, 0.05% NaN_3_ on a shaking platform at 4°C. Next day, after three PBST washing steps of 10min, specific HRP-tagged secondary antibodies, goat anti-mouse (GAM) at 1:50000 dilution; goat anti-rabbit (GAR) at 1:25000 dilution in PBST, were added to the membranes and incubated for 1h at RT under constant shaking. Blots were washed three times for 10min with PBST and images were taken with UVP imager using ECL SuperSignal™ West Dura (Pierce). Normalization and evaluation were performed using the UVP Software VisionWorks™ or Gelanalyzer. Blots were stripped with a mild stripping buffer (15g glycine, 1g SDS, 10mL Tween20, pH adjusted to 2.2 filled up to a total volume of 1L with ultrapure water) and re-probed with a different antibody.

### Pyroptosis Measurement by LDH Assay

Monocyte subpopulations (25000 cells) were treated with ATP (2mM) +/-LPS (500ng/mL) +/- MCC950(10μM) and incubated for a total time period of 45min. Cells were spun down, supernatant was transferred into a white, clear-bottom 384-well plate and kept overnight in the fridge for subsequent measurement of LDH release on a TECAN monochromatic plate reader.

### ELISA

Cell supernatants (50-100 µL equaling to 25,000-50,000cells/sample of monocytes from healthy subjects, CAD and ACS patients were used for measuring IL-1β, IL18, IL18BP and IL1RN release. Assays were performed according to the protocols described in the data sheets of the respective ELISA kit (see **Supplementary Table 2**). Final values were always calculated to pg/mL of 50000 cells in order to compare between assays.

### Chemicals and Antibodies

Reagents used incl. SYBR green primers and Taqman Assays are included in the **Supplementary Table 2**; antibody dilution can be provided upon request.

### Statistical Analysis

Statistical analysis was performed using GraphPad Prism 7. For experiments involving the three monocyte subpopulations in healthy subjects we used One-Way Anova with repeated measures (RM) and Tukey’s multiple comparisons test with individual variances computed for each comparison.

For experiments assessing monocyte subpopulations upon treatments to trigger rapid inflammasome activation both in healthy and cardiovascular disease patient samples we used Two-Way Anova with no pairing with Tukey’s multiple comparisons test. For comparing the means of two groups assuming differences in their variance we applied T-test with Welch correction. Unless stated otherwise data in bar graphs represents mean average and the error bars depict standard error of mean (S.E.M). The n-number represents number of independent biological samples and is noted in each figure legend. Statistical significance (p-value) was defined as follows: *** p ≤ 0.001; ** p ≤ 0.01; * p<0.05.

## Results

### Rapid Inflammasome Activation Is Attenuated During Early-Stage Myocardial Infarction

**Figure 1:**
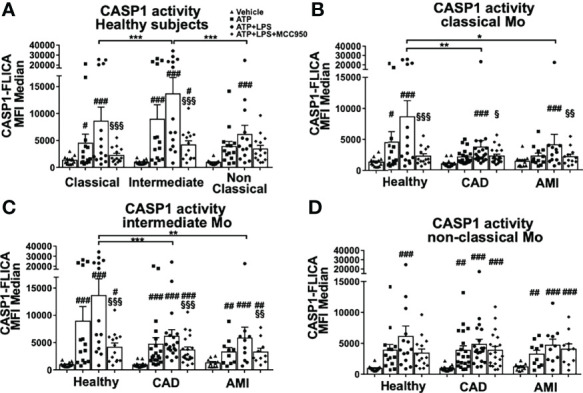
Rapid inflammasome activation is attenuated in monocyte subpopulations during AMI patients. Monocyte subpopulations simultaneously treated 45 minutes with 500ng/ml LPS and 2mM ATP, or ATP only, or in the presence of 10μM MCC950 were evaluated using CASP1 activation assay with FAM-YVAD-FMK peptide and flow cytometry measurement of median fluorescence intensity (MFI). **(A)** All three monocyte subpopulations from healthy donors induced CASP1 activation upon rapid inflammasome activation but only classical and intermediate monocytes showed MCC950-mediated inhibition. Intermediate monocytes showed the strongest CASP1 activation (n = 14). Measurements of CASP1 activity from **(B)** classical, **(C)** intermediate and **(D)** non-classical monocytes of healthy donors or CAD and AMI patients. Rapid inflammasome activation was significantly attenuated in classical and intermediate monocytes from CAD and AMI patients compared to healthy monocytes (n = 10-20). # *vs* vehicle and § *vs* LPS+ATP within same subpopulation and subject group; * comparison between disease groups; *, # or § p < 0.05; **, ## or §§ p ≤ 0.01; ***, ### or §§§ p ≤ 0.001.

Monocytes isolated from blood samples from healthy donors were FACS sorted into classical (CD14^++^CD16^-^), intermediate (CD14^++^CD16^+^) and non-classical (CD14^+^CD16^++^) subpopulations (**Figure S1A**). We induced rapid inflammasome activation of these monocytes by simultaneous treatment with LPS (500 ng/ml) and ATP (2mM) for a period of 45 min, which triggers transcriptional-independent activation of NLRP3 (20). The extent of inflammasome activation was determined by measurement of CASP1 activity with a fluorometric assay. In healthy donors, CASP1 activity was detectable in all three subpopulations upon treatment but was significantly higher in intermediate monocytes (**Figure 1A**). Parallel measurements of intracellular pro-CASP1 levels confirmed these results (**Figure S1B**). However, treatment with MCC950, a specific NLRP3 inhibitor, significantly reduced CASP1 activity only in classical and intermediate subsets (**Figure 1A**). Therefore, low levels of CASP1 activity detected in non-classical monocytes were regarded as NLRP3-independent and most likely not related to the rapid inflammasome activation investigated in this study.

Monocyte function and inflammasome activation have been implicated with numerous inflammatory diseases including cardiovascular disease. Next, we determined rapid inflammasome activation of monocyte subpopulations isolated from stable coronary artery disease (CAD, n=20) and acute myocardial infarction (AMI, n=10) patients. Both CAD and AMI patients displayed strong attenuation of CASP1 activity in classical and intermediate monocytes when compared with healthy donors (**Figures 1B–D**). Importantly, rapid inflammasome activation in CAD and AMI monocytes was only partially attenuated and could be further inhibited by MCC950. Of note, our data showed a high dispersion of CASP1 activity values which highlight a pronounced inter-individual variability. In fact, the group of healthy subjects presented with a group of high responders (n=6 out of 14 samples) while in CAD and AMI groups high responders were rather sporadic. In this respect, when comparing monocyte subpopulations (e.g., classical versus intermediate) or treatments (vehicle versus ATP+LPS) within the same subject group the use of paired statistical analysis partially compensates for this variability. However, statistical comparison between disease groups (e.g., healthy versus AMI) used unpaired analysis and prompts a careful interpretation of the results due to the high data dispersion and limited number of samples.

**Figure 2:**
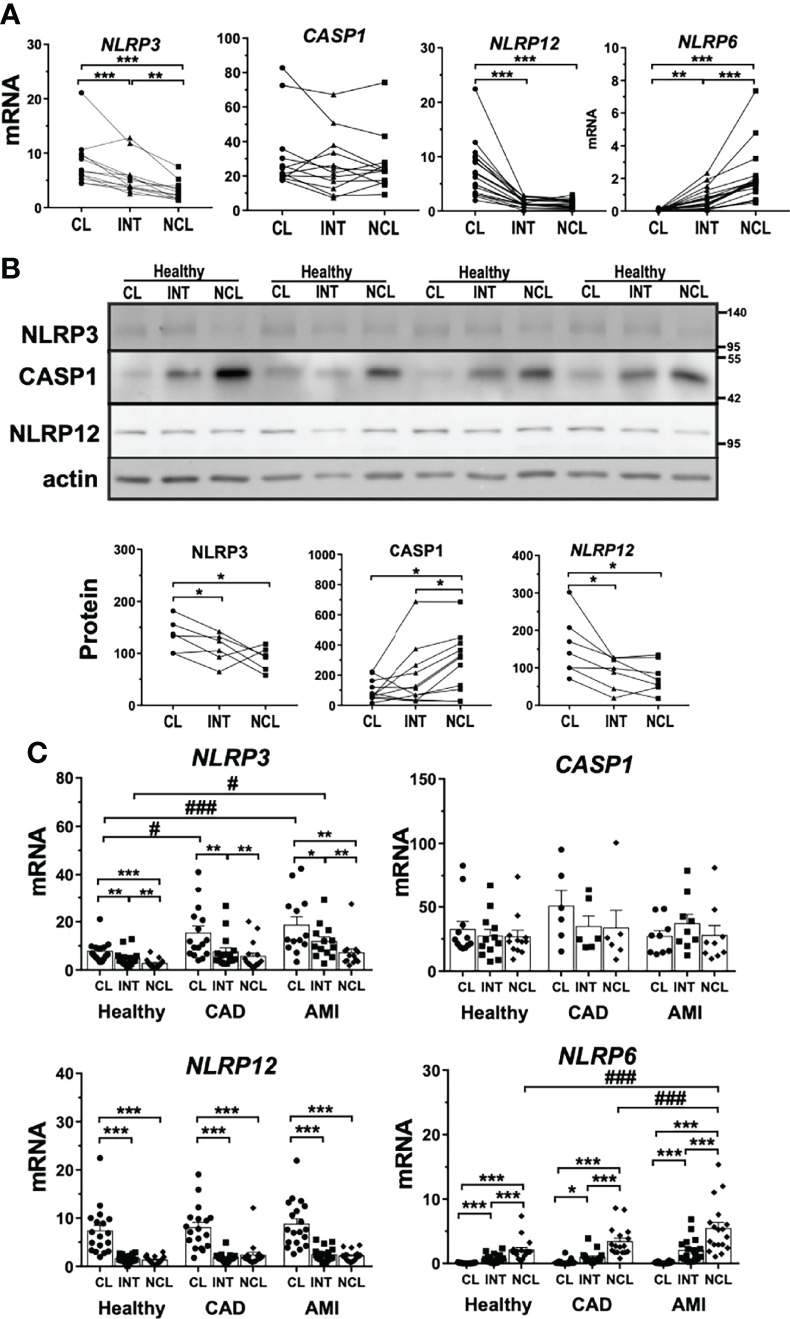
Basal expression of inflammasome-associated genes differs between human monocyte subpopulations. Basal expression of various inflammasome-associated genes in untreated monocyte subpopulations from healthy donors. **(A)** mRNA expression measured by qPCR (n = 8-15) and **(B)** protein levels by immunoblotting (n = 4-11) revealed striking differences for NLRP3 and 12, as well as CASP1 between subpopulations. **(C)** Comparing healthy to CAD or AMI monocytes revealed increased gene expression of NLRP3 in classical monocytes and of NLRP6 in non-classical monocytes of AMI patients; * compared to classical; # compared to healthy; * or # p < 0.05; ** or ## p ≤ 0.01; *** or ###p ≤ 0.001.

In order to better understand factors driving the observed differences in rapid inflammasome activation we evaluated basal expression patterns of main protein components of the inflammasome complex. NLRP3 is the most well characterized member of the NLR family and the major inflammasome suspected to be involved in rapid inflammasome activation in our assays. NLRP3 mRNA expression was higher in human classical monocytes following a stepwise decline along the other subpopulations (**Figure 2A**). Accordingly, NLRP3 protein levels showed a moderate reduction in non-classical monocytes reflecting a lower basal availability of this basic component of the inflammasome. Interestingly, NLRP3 mRNA expression levels from CAD and AMI patients both in classical and intermediate monocytes presented upregulation compared to healthy donors (**Figure 2C**). Despite increased NLRP3 expression, CAD and AMI subpopulations presented an attenuated CASP1 activity suggesting other factors involved in the regulation of inflammasome activation.

Other two NLR proteins, NLRP6 and NLRP12, have been reported to act as immune-modulatory agents by negatively regulating pivotal inflammatory pathways such as NF-κB signaling (31, 32). NLRP12 showed higher gene and protein expression in classical monocytes (**Figures 2A, B**). Contrarily, NLRP6 displayed negligible mRNA expression in classical monocytes but was expressed in intermediate and non-classical subpopulations (**Figure 2A**). Although differential expression of these two genes could partially explain the differences observed between monocyte subpopulations, it seems not to be linked to inflammasome attenuation in disease patients. NLRP12 expression was comparable in patients with CAD or AMI with preserved upregulation in classical monocytes but no increased expression during disease (**Figure 2C**). On the contrary, NLRP6 gene expression was strongly upregulated in AMI patients but exclusively in non-classical monocytes (**Figure 2C**). These findings suggest that neither NLRP6 nor NLRP12 might act as culprit regulators of rapid inflammasome activation during AMI.

Moreover, we evaluated the expression profile of inflammatory CASP1 as the main effector of inflammasome complex activation. Monocyte subpopulations showed comparable, although quite variable, CASP1 gene expression (**Figure 2A**). Surprisingly, CASP1 precursor protein (pro-CASP1) showed significantly lower expression in classical monocytes compared to the other subpopulations (**Figure 2B**). Additionally, we measured expression levels of P2RX7, a ligand-gated ion-channel responsible for ATP mediated triggering of inflammasome complex formation. P2RX7 mRNA expression was constant between monocyte subpopulations as well as compared with patient-derived monocytes (**Figures S2A, C**).

As in healthy donors, patient-derived non-classical monocytes also presented low levels of CASP1 activity that were insensitive to NLRP3 inhibition. Thus, we explored if other inflammasomes could be responsible for the minor CASP1 activity in non-classical monocytes. NLRP1 and NLRC4 inflammasomes, also expressed in human monocytes, were downregulated in non-classical monocytes (**Figure S2**). NLRC4 inflammasome requires an accessory protein called NAIP to trigger its activation. Non-classical monocytes displayed lower protein levels of both NLRC4 and NAIP compared to classical monocytes (**Figure S2B**). Therefore, basal expression levels of NLRP1 or NLRC4 inflammasomes cannot explain CASP1 activity in non-classical monocytes. Additional to CASP1 two other inflammatory caspases have been described in humans, namely CASP4 and CASP5. CASP4 showed no expression differences between monocyte subpopulations (**Figure S2A**). However, CASP5 expression was significantly higher in non-classical monocytes both at mRNA and protein levels, which could account at least partially for the signal measured in our CASP activity assay (**Figure S2**). CASP5 protein expression seems exclusively restricted to non-classical monocytes (**Figure S2B**) and therefore we expect it not to contribute to our CASP activity measurements in classical and intermediate monocytes. We also measured basal expression of these genes in monocyte subpopulations isolated from CAD and AMI patients and no significant differences were observed (**Figure S2C**).

### Rapid Inflammasome Activation Triggers Transcription Independent IL18 Release Primarily in Classical Monocytes

**Figure 3:**
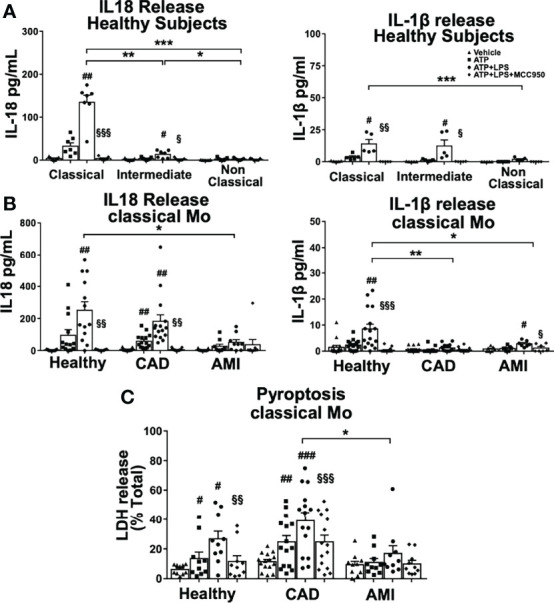
IL18 release is predominant in classical monocytes upon rapid inflammasome activation and is greatly attenuated in AMI patients. ELISA measurement of inflammatory cytokines IL18 and IL-1β released from monocyte subpopulations treated as described in **Figure 1**. **(A)** Classical monocytes from healthy donors showed clear predominance for IL18 release compared to the two other subpopulations (n=5-7). IL18 release was completely inhibited by MCC950 treatment. IL-1β was released at a much lower extent than IL18, but still significantly different between classical and non-classical monocytes. **(B)** Classical monocytes from AMI patients showed a significant impairment of both IL18 and IL-1β release. Decreased IL18 release was not observed in monocytes from CAD patients. **(C)** LDH release as a measure of pyroptotic cell death was significantly induced upon rapid inflammasome activation in classical monocytes of healthy subjects and CAD patients but substantially attenuated in monocytes from AMI patients. # *vs* vehicle; § *vs* LPS+ATP; *, # or § p < 0.05; **, ## or §§ p ≤ 0.01; ***, ### or §§§ p ≤ 0.001.

We have demonstrated a distinct rapid NLRP3 activation among monocyte subpopulations measured by CASP1 activity and most importantly a strong attenuation thereof in monocytes of CAD and AMI patients. This prompted us to measure proteolytic cleavage and release of inflammatory interleukins IL-1β and IL18 as the major outcome of inflammasome activation. First, we measured interleukin release from monocyte subpopulations of healthy subjects. Classical monocytes showed significantly increased release of IL18 after 45 minutes treatment and to a much greater extent than intermediate monocytes (**Figure 3A**). As expected, IL-1β release was minimal compared to IL18, in accordance with the requirement for transcriptional-dependent production of IL-1β. On the contrary, canonical inflammasome activation with overnight transcriptional priming of classical monocytes shifted the profile of interleukin release, with a 10-fold increase of IL-1β release and significantly lower IL18 when compared to rapid inflammasome activation (**Figure S3A**). Surprisingly, intermediate monocytes released very low levels of both IL18 and IL-1β despite high CASP1 activity and no release was observed in non-classical monocytes (**Figure 3A**).

Importantly, IL18 release from classical monocytes required licensing of NLRP3 since using only ATP without LPS priming induced minimal interleukin release (**Figure 3A**). In addition, NLRP3 inhibition by MCC950 treatment completely blocked interleukin release in a similar manner as the observed inhibition of CASP1 activity. Overall, rapid activation of the NLRP3 inflammasome induced predominantly acute IL18 release, which seemed to be restricted to classical monocytes in healthy donors.

Next, we measured interleukin release from classical monocytes obtained from acute and stable cardiovascular disease patients to assess if attenuation of rapid inflammasome activation affected IL18 production in monocytes from these patients. Indeed, mature IL18 release was dramatically diminished in classical monocytes from AMI patients (Fig, 3B). Surprisingly, IL18 release was not strongly reduced in classical monocytes from CAD patients (**Figure 3B**) although they showed attenuated CASP1 activity (**Figure 1B**). In contrast, the already low levels of IL-1β detected in healthy classical monocytes were further reduced in both AMI and CAD patients (**Figure 3B**). These data suggest an acute refractory state of circulating classical monocytes during the early inflammatory phase of myocardial infarction, which probably is transitory and may prevent excessive systemic release of IL18.

**Figure 4:**
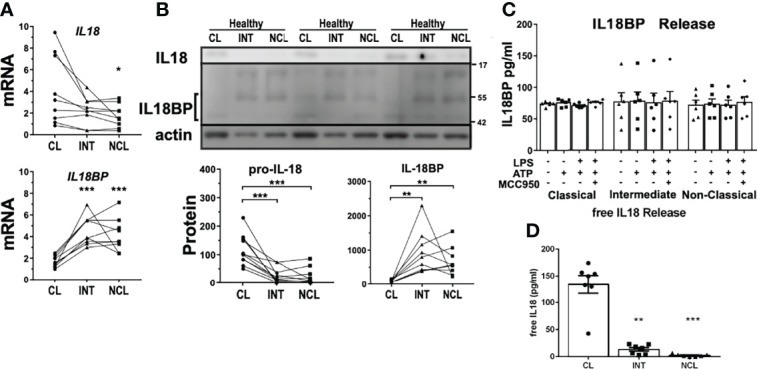
Differential expression profile of IL18 gene as a potential explanation for variation of inflammatory interleukin release. **(A)** Basal mRNA and **(B)** protein expression profiles of IL18 and IL18BP in monocyte subpopulations of healthy donors. IL18 expression was significantly higher in classical monocytes while intracellular glycosylated IL18BP protein levels (~45-50 kDa) were higher in intermediate and non-classical monocytes (n = 9-11). At least two bands were detected that would correspond to different IL18BP isoforms. **(C)** Unlike IL18, IL18BP release into the supernatant from monocyte subpopulations upon treatments, was unaffected by inflammatory stimulation (n = 6). **(D)** Therefore, active free IL18 using calculated IL18BP sequestration resulted in practically the same IL18 concentrations measured in **Figure 3A**; * compared to classical; *p < 0.05; **p ≤ 0.01; ***p ≤ 0.001.

As mentioned above, intermediate monocytes released very low levels of IL18 despite a strong CASP1 activity upon rapid inflammasome activation. Interestingly, *IL18* gene expression was slightly upregulated in classical monocytes relative to the other subpopulations (**Figure 4A**). However, western blot analysis indicated a much greater intracellular pool of pro-IL18 in classical monocytes compared to intermediate monocytes. The significantly reduced levels of constitutive pro-IL18 protein in intermediate subpopulation might partially explain the selective IL18 release by classical monocytes. Additionally, we evaluated the expression levels of IL18 binding protein (IL18BP), a physiological inhibitor of IL18 that modulates its extracellular availability and activity. Both mRNA (**Figure 4A**) and intracellular protein expression (**Figure 4B**) of IL18BP were greatly upregulated in intermediate and non-classical monocytes. IL18BP is a small protein of only 21 kDa in size but as it enters the secretory pathway it undergoes glycosylation which increases its molecular size to about 45-50 kDa (**Figure 4B**). In fact, IL18BP expression pattern was opposing to the one of IL18 in monocyte subpopulations. We then measured IL18BP release from monocyte subpopulations upon rapid inflammasome activation to evaluate the impact of these different expression profiles. Surprisingly, all monocyte subpopulations released equivalent IL18BP levels at steady state, no matter of their intracellular expression levels (**Figure 4C**). Moreover, IL18BP release was unaffected by inflammatory stimulation indicating that it is not triggered by rapid inflammasome activation. We wondered if basal IL18BP release could affect IL18 release as an autocrine factor modulating the interleukin activity. However, basal extracellular IL18BP levels released by monocytes are not high enough to properly block IL18 as shown by calculation of the effective free IL18 levels considering IL18BP dissociation constant (Kd=400nM) (**Figure 4D**). However, we cannot exclude some intracellular regulatory interaction between IL18 and IL18BP in their respective secretion pathways that could affect IL18 levels of intermediate monocytes.

In contrast to *IL18*, which is constitutively expressed by immune cells as well as a wide range of epithelial cells, *IL-1β* requires transcriptional priming (33). Although IL-1β does not play an important role in rapid inflammasome activation, *w*e also measured gene expression levels of *IL-1β*, related receptors IL1R1 and IL1R2 as well as IL-1β receptor antagonist (IL1RN) in monocyte subpopulations. Most of these genes showed lower expression in non-classical monocytes, including lower IL1RN released levels compared to classical monocytes (**Figures S3B, C**). Interestingly, IL1RN protein release was induced upon rapid inflammasome stimulus, unlike the release of IL18BP, both in classical and intermediate monocytes.

An additional feature of inflammasome activation is the induction of pyroptotic cell death downstream of CASP1 activation. Classical monocytes showed significantly increased levels of LDH release as one marker of pyroptosis being also inhibited by MCC950 highlighting the NLRP3 specificity of rapid inflammasome activation. The induction of LDH release was also attenuated in monocytes from AMI patients when compared with CAD and healthy subjects (**Figure 3C**). Interestingly, MCC950 was able to restore LDH levels in classical monocytes of CAD and AMI patients to the levels of ATP stimulation only. Of note, attenuation of pyroptotic LDH release from AMI monocytes was milder than the respective cytokine inhibition underlining a stronger refractory impact on the regulation of IL18 release.

### Potential Regulatory Proteins of Rapid Inflammasome Activation in Monocyte Subpopulations

Identification of a growing number of NLRP3 modulators highlights the complexity and fine-tuning that governs inflammasome activation. Determination of monocyte subpopulation-specific profiles of known inflammasome modulators may result in a better insight of particular regulatory mechanisms explaining the diverse inflammasome activation in monocyte subpopulations and disease stages.

**Figure 5:**
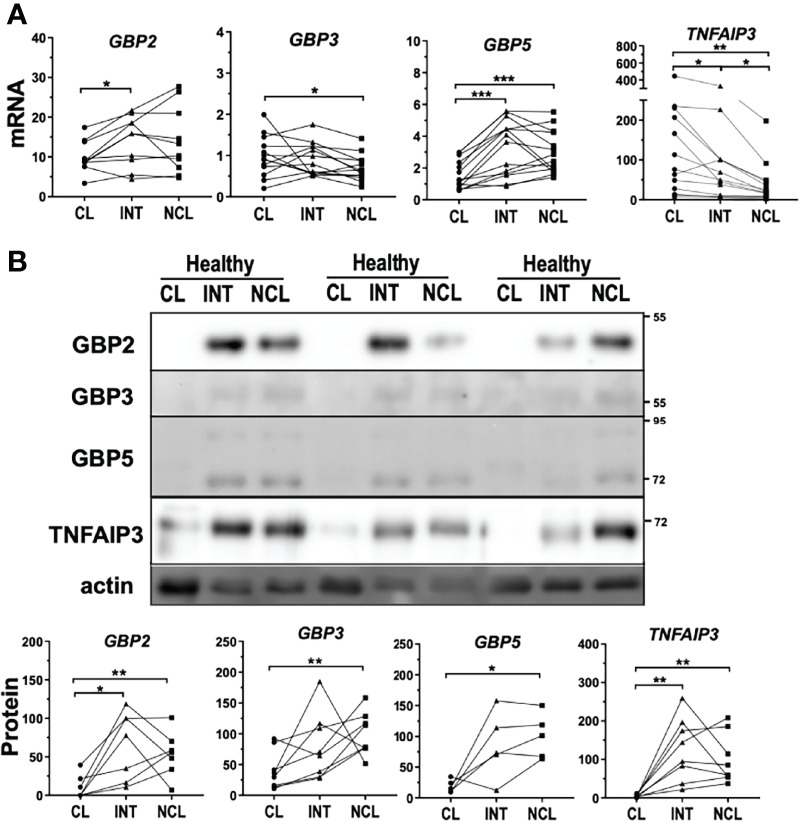
Differential expression levels of genes regulating inflammasome activation between monocyte subpopulations. **(A)** mRNA expression and **(B)** protein levels of GBP2, 3 and 5 displayed downregulation in classical monocytes from healthy donors more significantly at protein level. TNFAIP3 had significantly higher mRNA expression in classical monocytes but TNFAIP3 protein levels were higher in both intermediate and non-classical monocytes; * comparison between subpopulations. *p < 0.05; **p ≤ 0.01; ***p ≤ 0.001.

Guanylate binding proteins (GBP) have been shown to modulate inflammasome activation including a direct role of GBP5 in NLRP3 oligomerization (34) as well as an involvement of GBP2/3 in non-canonical inflammasome activation (35). Analysis of mRNA and protein expression showed markedly reduction of GBP5 in classical monocytes compared to the other subpopulations (**Figures 5A, B**). Of note, GBP5 is a positive modulator of NLRP3 and lower expression of GBP5 in classical monocytes would not explain the high inflammasome activation and IL18 release shown in this subpopulation. *GBP2* and *3* mRNA expression was mostly unchanged between subpopulations with just minor differences between subpopulations (**Figure 5A**). At the protein level, both GBP2 and GBP3 were only significantly increased in non-classical monocytes. In a similar way to *GBP5*, mRNA and protein expression of GBP1 and 4 were also diminished in classical monocytes (**Figures S4A, B**). Although these two GBP proteins have been related to immune responses against intracellular pathogens, they have not been described to affect inflammasome activation. These findings suggest that GBP proteins might not be associated with rapid inflammasome activation in monocytes.

Bruton’s Tyrosine kinase (BTK), another mediator of inflammasome activation, has been shown to induce inflammasome activation by direct phosphorylation of NLRP3 (36) and was higher expressed in non-classical monocytes of healthy donors (**Figure S4A**). In contrast, mRNA expression of TNF-alpha induced protein 3 (*TNFAIP3)* was significantly increased in classical monocytes compared to the other subpopulations (**Figure 5A**). TNFAIP3, also known as A20, is a key regulator of TLR signaling acting as a negative feedback regulator of NF-kB pathway (37). Remarkably, TNFAIP3-deficient mice exhibit spontaneous NLRP3 activation suggesting that TNFAIP3 action can modulate inflammasome activity presumably by posttranslational modifications (38). Interestingly, TNFAIP3 protein levels were profoundly diminished in classical monocytes compared to the other subpopulations in healthy subjects (**Figure 5B**).

The inverted relationship between mRNA and protein levels might suggest strong post-transcriptional regulation of TNFAIP3 within the subpopulations.

### TNFAIP3 Is Upregulated During the Early Stage of Acute Myocardial Infarction

**Figure 6:**
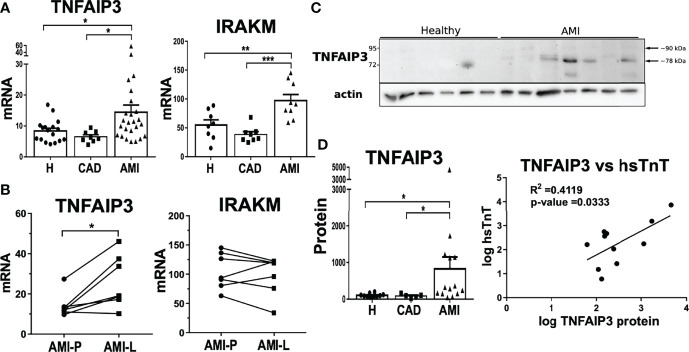
Upregulation of negative feedback loop regulators TNFAIP3 and IRAKM linked to attenuated inflammatory phenotype in AMI CD14^+^ monocytes. **(A)** Both TNFAIP3 and IRAKM mRNA was significantly increased in circulating CD14^+^ monocytes from AMI patients compared to healthy subjects or CAD patients (n = 8-27). **(B)** In contrast to IRAKM, the mRNA expression of TNFAIP3 was even further upregulated in lesional monocytes compared to peripheral cells within the same AMI patients (n = 7). **(C)** TNFAIP3 protein levels were also significantly higher in monocytes from AMI patients. **(D)** Moreover, TNFAIP3 protein showed a direct correlation with high sensitive Troponin T (hsTnT) levels. *p < 0.05; **p ≤ 0.01; ***p ≤ 0.001.

TNFAIP3 is a ubiquitin-editing enzyme that possesses both deubiquitinase (DUB) and ubiquitin E3 ligase activities, which is rapidly induced and acts as a negative feedback loop modulator. We therefore explored further if TNFAIP3 expression levels were upregulated during AMI, which might partially explain the attenuation of rapid inflammasome activation. Indeed, *TNFAIP3* mRNA expression was significantly upregulated in monocytes from AMI patients compared to healthy controls and CAD patients (**Figure 6A**). Additionally, we were able to compare monocytes collected during catheterization at the lesion area in the coronary artery (AMI-L) with peripheral blood monocytes collected at the catheter entry vessel (AMI-P) of the same subject. Lesional monocytes presented a further increase of *TNFAIP3* mRNA expression in comparison to peripheral blood monocytes (**Figure 6B**). IRAKM expression was also significantly increased in CD14^+^ monocytes from patients with AMI compared to both healthy and CAD subjects (**Figure 6A**). IRAKM is also a negative regulator of TLR and NF-kB signaling that can act as a negative feedback loop in monocytes (39). In contrast to TNFAIP3, IRAKM expression did not differ between lesional and peripheral monocytes from AMI patients (**Figure 6B**). Of note, IRAKM transcriptional induction is much slower than TNFAIP3 as we demonstrated in the THP1 monocytic cell line. Upon LPS treatment, TNFAIP3 expression was rapidly induced after 2h whereas significant upregulation of IRAKM was delayed until 4-6h with a maximum expression at 12h of treatment (**Figure S5A**). The fact that TNFAIP3 expression is stronger at the culprit lesion suggests that various factors triggering its upregulation could be associated either with the thrombotic event or with the subsequent ischemic cellular damage. In both cases, the release of DAMPs can activate TLR receptors on the surface of monocytes, which also induces the transcriptional and post-transcriptional upregulation of TNFAIP3. In a similar way, the severity of the ischemic lesion or the time lapse between ischemic event and sample collection at percutaneous coronary intervention (PCI) can strongly influence monocyte activation by DAMPs. As a result, TNFAIP3 expression levels present a big inter-subject variability.

Using CD14^+^ monocytes in this experiment, we were able to obtain enough material to measure TNFAIP3 protein expression. Protein levels of TNFAIP3 were significantly higher in AMI patients than in healthy controls (**Figure 6C**). By comparison, IRAKM protein levels were not markedly different between healthy and AMI patients but were highly variable between individuals (data not shown). Interestingly, TNFAIP3 protein in AMI-derived monocytes displayed two protein bands of different molecular size. The upper one corresponds to the full length TNFAIP3 (89.9 kDa), and a smaller protein around 78 kDa, which predominated in monocytes from patients (**Figure 6C**). Contrarily, THP1 cells expressed both full length and short TNFAIP3 proteins but TLR stimulation only induced upregulation of the full-length molecule (**Figure S5B**).

Intriguingly, TNFAIP3 protein levels of monocytes from AMI patients showed a mild positive correlation with circulating levels of high sensitive cardiac troponin T (hsTnT) levels (**Figure 6D**). Since cardiac troponin levels are the gold standard to determine myocardium damage, this result implies a link between the severity of tissue injury and TNFAIP3 upregulation. Certainly, troponin does not directly trigger inflammatory signaling pathways but its release from cardiomyocytes is paralleled by release of DAMPs from the same damaged cells. As mentioned previously, DAMPs are able to activate TLR signaling and trigger TNFAIP3 production. In fact, this process would be similar to the tolerance induced by LPS pre-stimulation that upregulates TNFAIP3 as a negative feedback factor driving the inhibition of NF-kB signaling pathway. This is shown in THP1 by preventing p65 phosphorylation and increased levels of negative regulator IkBα (**Figure S5B**).

Overall, rapid upregulation of TNFAIP3 in monocytes during the acute phase of AMI may not only lead to a negative regulation of NF-kB activation but also to a non-transcriptional attenuation of rapid NLRP3 inflammasome activation and a concomitant reduction of IL18 release. All these findings highlight the importance of spatiotemporal regulation of inflammasome activation and the fine-tuning mechanisms that keep inflammasome activation in monocyte subpopulations in check.

## Discussion

The innate immune system is a crucial first responder against external pathogens but plays also a critical role in restoring homeostasis after sterile tissue injury. In the onset of AMI, ischemia and ischemia-reperfusion (I-R) induces cardiac cell death that leads to a strong inflammatory response and a massive recruitment of myeloid cells. Among these cells, we are especially interested in the role of monocytes as first responders and in this study, we addressed monocyte capabilities to trigger rapid inflammasome activation in the onset of AMI.

Cardiac injury is accompanied by NLRP3 inflammasome activation within immune cells recruited to the lesion (40) as well as in other tissue resident cells within the ischemic area (41). Experimental data in animal models have also shown that inflammasome activation causes further myocardial damage and that NLRP3 inhibition reduces infarct size and preserves cardiac function (42). Canonical NLRP3 inflammasome activation requires two signals: priming by TLR activation with DAMPs released from damaged cells (43, 44) and a trigger signal that in sterile injury relies on high levels of extracellular ATP.

Most studies have focused on transcriptional inflammasome activation where priming lasts several hours (enough time to induce *de novo* synthesis of inflammasome components) before the trigger stimulus. Conversely, rapid inflammasome activation occurs when priming and trigger signals occur simultaneous or within a short period of time causing a transcription independent activation mostly driven by post-translational modifications. Our study defined for the first time rapid inflammasome activation of human monocyte subpopulations both in healthy subjects as well as in CAD and AMI patients.

In healthy subjects, non-classical monocytes displayed the lowest CASP1 activity and reduced sensitivity towards the NLRP3 specific inhibitor MCC950 (45). Therefore, rapid inflammasome activation seems restricted to classical and intermediate subpopulations. However, despite higher CASP1 activity in intermediate monocytes, rapid inflammasome activation triggered IL18 release almost exclusively in classical monocytes. Of note, CASP1 activity signal presented with high inter-individual variability with a significant group of high responders within the healthy subjects. Some individuals might be more prone to higher rapid inflammasome response, but at this point we cannot assess which factors determine this feature. IL18 release, instead of IL-1β, is characteristic for rapid inflammasome activation due to the basal expression of its precursor (pro-IL18), which does not require *de novo* transcriptional synthesis. Although pro-IL18 is considered to be constitutively expressed in monocytes and macrophages (33), our analysis revealed that classical monocytes expressed much higher pro-IL18 protein levels. In contrast, IL18 mRNA was expressed at a similar rate in all subpopulations suggesting strong post-transcriptional mechanisms in intermediate and non-classical monocytes. Post-transcriptional regulation has been reported to modulate mRNA stability and translation rate of several interleukins, including IL-1β (46), but to the best of our knowledge has not yet been described for IL18.

**Figure 7:**
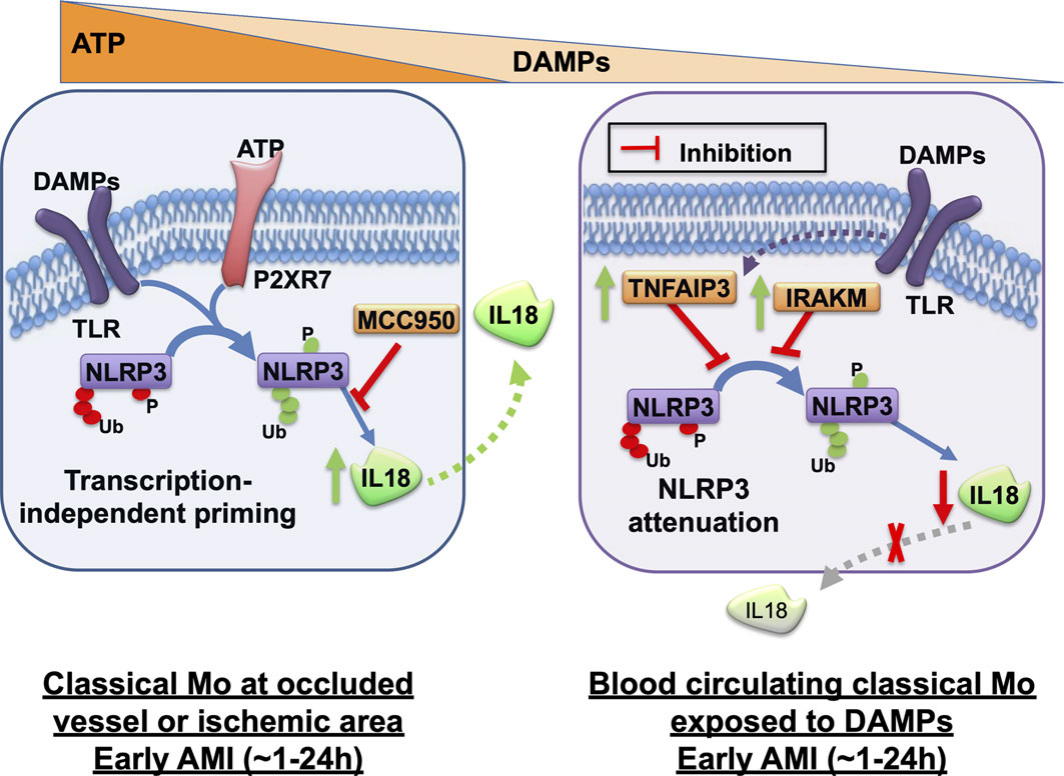
Graphical summary of rapid inflammasome activation events in monocytes during acute myocardial infarction. Rapid inflammasome activation happens after simultaneous dual signal, DAMP-induced TLR priming and extracellular ATP sensing by P2RX7 at the injury site. TLR signaling triggers removal of ubiquitin modification or phosphorylation (red tags) of basal NLRP3 that repress inflammasome complex formation. The same signaling cascades also induces post-translational modifications in other residues that enhances inflammasome activation (green tags). P2RX7 ion-channel activation triggers intracellular K+ efflux, which induces NLRP3 multimerization. Active inflammasome complex recruits pro-CASP1, its catalytic activation and subsequent pro-IL18 cleavage and release. Rapid inflammasome activation and IL18 release happens in classical monocytes (Mo) at the site of the occluded vessel or in the ischemic area at the early phase of AMI, within the first hours after infarction (left panel). This pathway releases very low levels of IL-1β because IL-1β is transcription dependent. NLRP3 specific inhibitor, MCC950, can block this process. Circulating monocytes in the blood away from the site of ischemic lesion are potentially only able to sense DAMPs but not the short-lived ATP. Activation of only TLR signal induces upregulation of the anti-inflammatory mediators TNFAIP3 and IRAKM. TNFAIP3 potentially modulates a concomitant decline of inflammasome priming alongside a significant decrease of acute IL18 release in a protective mechanism against systemic inflammatory hyper-activation (right panel). The given time frames in this graphical summary are just an estimated guess of the authors.

Interestingly, we also encountered dramatic differences between monocyte subpopulations for the expression of IL18-binding protein (IL18BP), a physiological regulator of IL18. Whereas all subpopulations released equal amounts of IL18BP, intermediate and non-classical monocytes showed greater mRNA expression and intracellular protein levels relative to classical monocytes. Yet, the protein profile of IL18BP from intermediate and non-classical monocytes displayed prevalent higher molecular weight isoforms potentially attributable to post-translational modifications. It is not clear at this time if IL18BP could be linked to the control of intracellular IL18 levels or IL18 release in monocyte subpopulations. Our results indicate that classical monocytes are a main source of active IL18 release after simultaneous encounter with the two signals that trigger rapid inflammasome activation. A first pro-inflammatory wave of myeloid cells is required to clear necrotic and apoptotic cells within the site of ischemic injury. Although in our study we could not directly measure CASP1 activity in the cardiac lesion, we propose that rapid inflammasome activation is potentially occurring to classical monocytes recruited during this first wave (**Figure 7**, left panel). Classical monocytes in this context will release significant amounts of IL18 in the infarcted area and its border zone but will eventually also undergo pyroptotic cell death releasing additional DAMPs (e.g., S100A proteins).

The role of IL18 in AMI has not been clearly defined but growing evidence supports an important role of IL18 in both AMI and heart failure (47). Cooperative action of IL18 and IL12 (or IL15) induces interferon-γ (IFN-γ), which plays a relevant role in Th1 response. IL18 not only promotes atherosclerosis progression through IFN-γ-dependent inflammation within the lesion of animal models but may also trigger other pro-inflammatory functions independent of Th1 response including priming of human neutrophils for free radical production, cardiac depressant effects and contractile dysfunction.

Our study found evidence of a strong attenuation of rapid inflammasome activation and a significant reduction in mature IL18 release in circulating classical monocytes isolated from AMI patients directly at the time of PCI. Although counterintuitive, this attenuated phenotype of circulating monocytes might depict an adaptive mechanism to prevent persistent systemic inflammatory response. In fact, previous studies reported a similar attenuated phenotype for canonical NLRP3 activation (transcription dependent priming) in monocyte-derived macrophages obtained from stroke or AMI patients that was accompanied by decreased IL-1β release (29, 48).

In this respect, IL18 signaling not only triggers beneficial positive effects/functions, as described above, but has been also associated with deleterious outcomes Several studies have shown that IL18 can trigger apoptosis in endothelial cells, induce proliferation of cardiac fibroblast with subsequent fibrotic remodeling or depress cardiomyocyte contractility (42). Moreover, IL18 blockade with heterologous IL18BP or IL18 blocking antibodies was able to reduce infarct size, reduce myocardial inflammation and improve cardiac function in several animal models of AMI (49, 50). Indeed, plasma levels of IL18 in AMI patients are an independent predictor of increased risk of cardiovascular events and worse prognosis (51).

Therefore, this attenuated phenotype of circulation monocytes that restricts IL18 release might be rather a beneficial response to modulate IL18 action at later stages of myeloid recruitment to the ischemic lesion. Although there is some parallelism with the “exhausted” monocyte phenotype described after repeated exposure to LPS (as it happens in sepsis) that also display low cytokine release but high ROS production (52), we considerate that the attenuated phenotype that we observe in AMI maybe represents a beneficial physiological differentiation rather than a maladaptive extreme suppression of monocyte activity.

Inflammasome activation is a tightly regulated process and numerous modulators have been identified in the last decade. For example, guanylate binding proteins (GBP) such as GBP5 and GBP2/3 have been regarded as regulators of inflammasome activation (34, 35). Although we showed higher GBP2, GBP3 and GBP5 protein levels in intermediate or non-classical compared to classical monocytes none of these differences seemed plausibly associated to the rapid inflammasome activation examined in our study. Direct posttranslational modifications of inflammasome components, such as phosphorylation (53) or ubiquitination (20, 54), also modulate NLRP3 activation. In this respect, deubiquitinating enzymes (DUBs) can modulate inflammasome activity through direct modification of NLRP3 as previously shown by using ubiquitin isopeptidase inhibitor G5, which unspecifically inhibits a wide range of DUBs (55). We also observed strong inhibition of rapid inflammasome activation upon G5 treatment in human monocyte subpopulations (data not shown). BRCC3, for example, regulates NLRP3 activity by removing NLRP3 ubiquitin modifications that repress inflammasome oligomerization (55). Moreover, TNFAIP3 has been shown to negatively regulate inflammasome activation in bone marrow derived macrophages, in a process that requires TNFAIP3 association to a CASP1-containing complex (38, 56). TNFAIP3 is a dual ubiquitin-modifying enzyme originally identified as a protective factor against TNF-induced cytotoxicity (57) and master modulator of NF-kB signaling (58). TNFAIP3 has DUB activity that catalyzes the removal of K63 ubiquitin in numerous proteins that, for example, affect TLR signaling (59).

We showed differences in TNFAIP3 basal expression between monocyte subpopulations of healthy subjects with significantly higher *TNFAIP3* mRNA in classical monocytes. Surprisingly, TNFAIP3 protein levels where much lower in classical monocytes than in intermediate and non-classical monocytes. These results suggest that intermediate and non-classical monocytes have a basal pool of TNFAIP3 protein available, but classical monocytes might require *de novo* translation of this regulator. Furthermore, TNFAIP3 gene and protein expression were strongly upregulated in circulating CD14^+^ monocytes from AMI patients (**Figure 7**, right panel).

Although TNFAIP3 upregulation could reflect a negative feedback loop that partially explain inflammasome attenuation in monocytes from AMI patients, other potential mediators involved in the fine-tuning of rapid inflammasome activation cannot be excluded. In fact, we also measured the expression of IRAKM, a monocyte/macrophage specific regulator, which is associated to post-infarction remodeling since IRAKM knockdown lead to adverse remodeling and systolic dysfunction after AMI (60). IRAKM gene expression was upregulated in monocytes isolated from AMI patients, but several features of IRAKM expression in monocytes differ to TNFAIP3. First, a delayed upregulation of IRAKM in THP1 cells treated with LPS, highlighting a faster regulatory pace of TNFAIP3. Additionally, monocytes obtained from the occluded vessel (culprit lesion) during PCI showed higher TNFAIP3 expression than in peripheral circulating monocytes, while IRAKM expression was not different between lesional and peripheral monocytes. Lastly, IRAKM has not been directly associate to inflammasome activation in previous studies, but rather acts as a modulator of TLR signaling cascades (39).

Although we cannot corroborate it with our data, we think that DAMPs released from the tissue injury acts as the stimuli to induce the attenuated phenotype observed in AMI circulating monocytes. This statement is partially supported by the higher TNFAIP3 expression measured in monocytes on the occluded vessel (culprit lesion) but also because we observed a direct correlation between TNFAIP3 expression and cardiac injury. We detected a positive correlation between high sensitive troponin T (hsTnT), the gold standard of cardiomyocyte cell damage, and TNFAIP3 protein levels in circulating monocytes from AMI patients. This implies a potential connection between severity of cardiac tissue injury and attenuated rapid inflammasome activation, presumably through increased monocytic TNFAIP3 expression. Interestingly, this association was restricted to TNFAIP3 protein expression, but not to gene expression (data not shown) strengthening the idea of fast post-transcriptional induction of this protein in classical monocytes, as proposed above.

Although we consider DAMPs stimulation to cause the observed attenuated phenotype in circulating monocytes, we cannot exclude other factors acting either independently or synergistically with DAMPs. For one side, glucocorticoids, produced as a response to sterile tissue injury (61), are known to induce both TNFAIP3 and IRAKM expression. On the other side, cholinergic receptor nicotinic alpha 7 (CHRNA7) signaling stimulated by C-reactive protein (CRP) or alpha-1-antitrypsin(A1AT) have been proposed as an inhibitory mechanism of inflammasome activation (62, 63).

The main limitation of our study is the restricted number of patient samples, which we were able to recruit. As mentioned above, CASP1 activity measurements revealed high inter-individual variability with a group of high responders mostly observed for the healthy subjects. If there are certain individuals more predisposed to higher rapid inflammasome response and which factors (e.g., genetics, age or disease severity) can be associated to this group of high responders are pending questions that should be addressed in future studies. Another limitation is that we only evaluated a single collection time point for AMI patients at the most acute possible moment (during percutaneous intervention).

It would be reasonable to study further time points to include other stages of inflammatory response (e.g., inflammatory *vs* resolution) and determine the duration of the attenuated monocyte state. Moreover, although we believe TNFAIP3 plays a relevant role in the observed attenuated phenotype of monocytes from AMI patients, we are aware that it is only a partial mechanism, which probably depends on other mediators and demands further research.

It is paramount to define the precise mechanisms that trigger and modulate rapid inflammasome activation in monocytes to better tackle early inflammatory responses during AMI and reduce cardiac tissue damage leading to heart failure. Pharmacological modulation that inhibits inflammasome activation in acute phases of AMI might be a desirable approach in patients with elevated plasma IL18. These AMI patients might present with defective or limited generation of the described attenuated monocyte phenotype driving excessive IL18 release with deleterious consequences in cardiac healing. In this sense, we showed that MCC950 or other inhibitors of NLRP3 are able to reduce rapid inflammasome activation. These types of inhibitors have shown therapeutic potential in different small and large animal models of cardiovascular injury (25, 64). The application of such inhibitors in human AMI patients seems promising but demands further evaluation to better understand the underlying mechanisms.

## Data Availability Statement

The raw data supporting the conclusions of this article will be made available by the authors, without undue reservation.

## Ethics Statement

The studies involving human participants were reviewed and approved by the ethics committee in Zurich (Kantonale Ethik-Kommission Zürich (KEK)), Switzerland (KEK-ZH-Nr. 2012-0321) and the Berlin State Ethics Committee in Berlin, Germany (EA4/122/14 and EA1/270/16). The patients/participants provided their written informed consent to participate in this study.

## Author Contributions

Conceptualization: AK, HG, and UL. Methodology: AK, HG, AA, VF, MiM, and DJ. Sample recruitment: MaM, KK, JH, RK, PJ, LL, DL, NK, CS, DM, FZ, and AH. Original draft: AK and HG. Review and editing: AK, HG, VF, AA, TZ, SB, NK, TL, and UL. Funding acquisition: UL, TL, AK, and AA. All authors have read and discussed the manuscript. All authors contributed to the article and approved the submitted version.

## Funding

This study received funding from a Novartis research grant. The funder was not involved in the study design, collection, analysis and interpretation of data, the writing of this article or the decision to submit it for publication. The work was also supported by grants from Leducq, SNF (310030_149990, 31030_166576, SPUM 33CM30-124112 and 32473B_163271) as well as the DZHK (German Centre for Cardiovascular Research) (81Z2100202) and the Berlin Institute of Health (BIH).

## Conflict of Interest

The authors declare that the research was conducted in the absence of any commercial or financial relationships that could be construed as a potential conflict of interest.

## Publisher’s Note

All claims expressed in this article are solely those of the authors and do not necessarily represent those of their affiliated organizations, or those of the publisher, the editors and the reviewers. Any product that may be evaluated in this article, or claim that may be made by its manufacturer, is not guaranteed or endorsed by the publisher.

## Supplementary Material

Supplementary Figure 1 Gating strategy for fluorescence assisted cell sorting **(A)** PBMCs isolated from fresh EDTA blood and magnetically pre-sorted to obtain CD14^+^ monocytes were labeled with CD14 (APC) and CD16 (Percp-Cy5.5). First, we gated based on their forward and side scatter (P1). Then singlets were selected using both forward and side scatter width versus height (FSC-W vs FSC-H and SSC-W vs SSC-H) leading to population P2 and P3. The selection for the three monocyte subpopulations was set by gating for CD14-APC and CD16-PercpCy5.5 and defined as classic (CD14^++^CD16^-^), intermediate (CD14^++^CD16^+^) and non-classic (CD14^+^CD16^++^). **(B)** Western blotting was used to analyse intracellular pro-CASP1 expression in total lysates. Dual activation of inflammasome with ATP+LPS lead to a decreased expression of the pro-form of Caspase 1 (CASP1) in all three subpopulations, whereas NLRP3 inhibitor MCC950 was only capable of fully blocking its processing in classical monocytes.

Supplementary Figure 2 Expression profiles of other NLR inflammasome proteins and inflammatory caspases in human monocyte subpopulations. **(A)** Whereas the gene expression of P2RX7, CASP4 and NLRC4 showed no changes between subpopulations, NLRP1 and NAIP mRNA expression were significantly higher in classical monocytes. Non-classical monocytes displayed a strong up-regulation of CASP5. **(B)** Protein levels confirmed increased expression of CASP5 as well as decreased expression of NAIP in non-classical monocytes. However, NLRC4 protein levels were also decreased, although was not changed on mRNA expression. **(C)** We did not observed any significant changes of mRNA expression between disease groups compared to healthy subjects. *p <0.05; **p ≤0.01; ***p ≤0.001.

Supplementary Figure 3 The interleukin release profile shifts between canonical and rapid inflammasome activation. **(A)** A profile shift of inflammatory interleukins released from rapid (acute – 45min) to transcription-dependent inflammasome activation (overnight-16h priming with LPS and then next ATP activation) was observed in classical monocytes. Higher IL18 was secreted in response to rapid activation whereas IL-1β predominates upon canonical priming involving transcription of necessary protein components (n=3-6). **(B)** Expression of IL-1β-associated genes such as IL-1β, IL1R1 and IL1R2 as well as IL1RN varies significantly within monocyte subpopulations, presenting a higher basal expression in classical (CL) versus non-classical (NCL) monocytes. IL-1β itself was significantly lower expressed in NCL monocytes, and very variable in the other two subpopulations (n=5-12). **(C)** Release of interleukin 1 receptor antagonist (IL1RN) is significantly increased in monocyte subpopulations to a different extent upon dual inflammasome activation.Classical monocytes show a significant higher release of IL1RN upon inflammasome activation than non-classical monocytes. The release was not inhibited by MCC950 incubation (n=3-13). * vs classical; § vs intermediate;*p <0.05; **p ≤0.01; ***p ≤0.001.

Supplementary FIgure 4 Basal expression analysis of other NLRP3 regulatory proteins. **(A)** mRNA expression of GBP1 was significantly higher expressed in intermediate monocytes and GBP4 as well as BTK show a significant increased in non-classical monocytes. **(B)** The protein levels of both GBP1 and GBP4 displayed an increase in intermediate and in non-classical monocytes as we showed for the other members of the family. *, p <0.05; ** p ≤0.01; *** p ≤0.001.

Supplementary FIgure 5 Transcriptional upregulation of TNFAIP3 upon TLR stimulation is faster than IRAKM expression in a timeline that would fit tolerance induction in AMI patients. **(A)** Time course of LPS (100ng/mL) induced gene expression profile of TNFAIP3 and IRAKM in THP1 cells. **(B)** Refractory behaviour induced by LPS denoted by decreased p65 phosphorylation and increased IkB-alpha levels in THP1 parallels TNFAIP3 protein upregulation. * vs LPS treatment; *p <0.05; **p ≤0.01; ***p ≤0.001.

Supplementary Table 1 Baseline characteristics of healthy subjects, patients with stable coronary artery disease (CAD) and acute myocardial infarction (AMI)

Supplementary Table 2 Key resource table (KRT) containing chemicals, antibodies, primers and kits used within the manuscript.

## Abbreviations

CD14^++^CD16^-^, classical monocytes; CD14^++^CD16^+^, intermediate monocytes; CD14^+^CD16^++^, non-classical monocytes; NLR, Nucleotide-binding Oligomerization Domain or NOD-like receptor; NLRP, NLR and Pyrin domain containing; NLRC4, NLR and CARD Domain Containing 4; CASP, caspase; IL-1β, interleukin-1β; IL18, interleukin-18; IL18BP, interleukin 18 binding protein; IL1RN, interleukin 1 receptor antagonist; AMI, acute myocardial infarction; CAD, stable coronary artery disease; GBP, guanylate binding proteins; TNFAIP3, TNF Alpha Induced Protein 3 (aka A20); IRAKM, IL-1 Receptor-Associated Kinase M aka IRAK-3, Interleukin 1 Receptor Associated Kinase 3; PCI, percutaneous coronary intervention.
