# Supplementary figures and images for: Rapid Inflammasome Activation Is Attenuated in Post-Myocardial Infarction Monocytes
Source: Front Immunol. 2022 Apr 26;13:857455. doi: 10.3389/fimmu.2022.857455 (PMC9090500; doi:10.3389/fimmu.2022.857455)

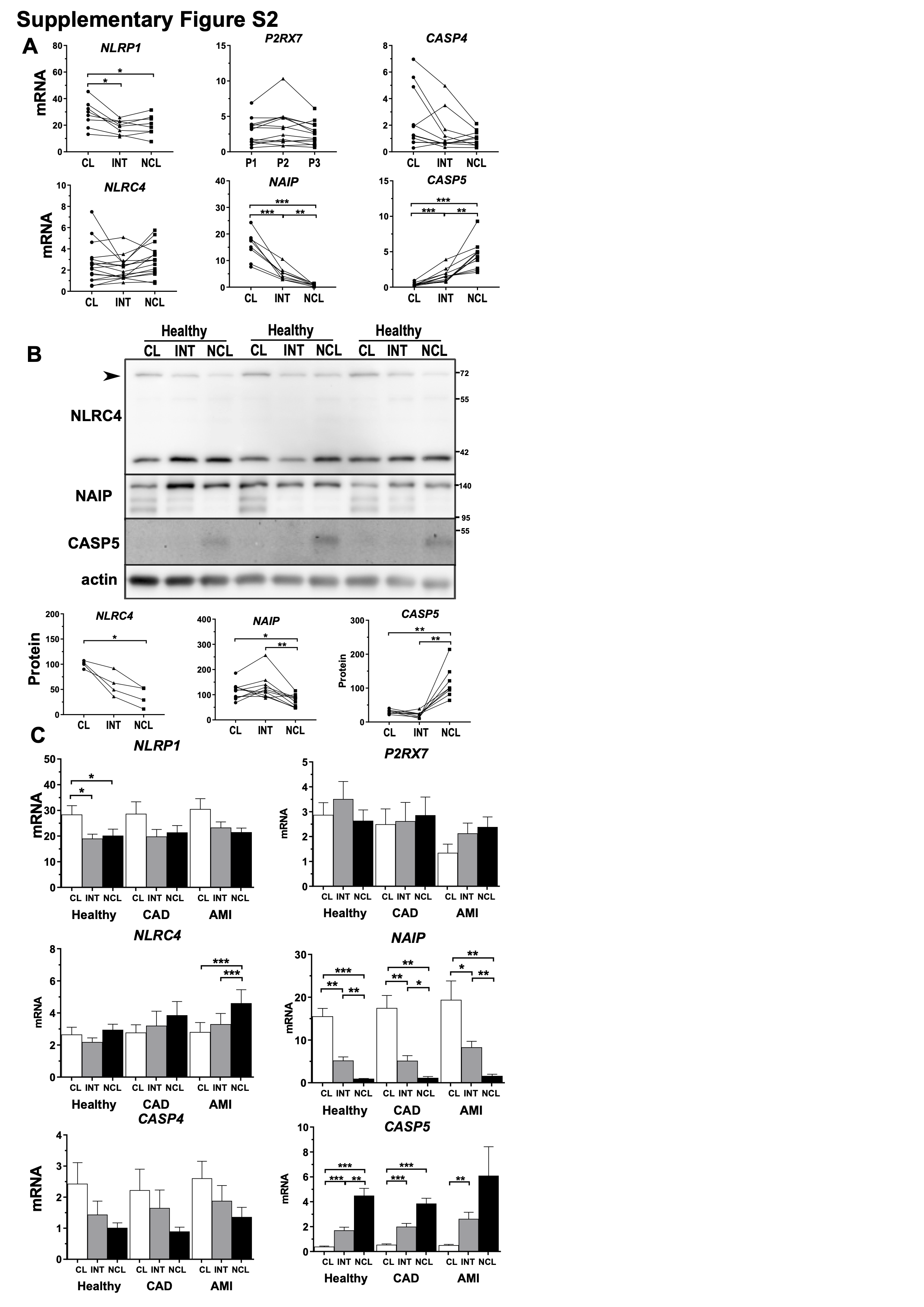

Supplement: Supplementary Figure 2 — Expression profiles of other NLR inflammasome proteins and inflammatory caspases in human monocyte subpopulations. (A) Whereas the gene expression of P2RX7, CASP4 and NLRC4 showed no changes between subpopulations, NLRP1 and NAIP mRNA expression were significantly higher in classical monocytes. Non-classical monocytes displayed a strong up-regulation of CASP5. (B) Protein levels confirmed increased expression of CASP5 as well as decreased expression of NAIP in non-classical monocytes. However, NLRC4 protein levels were also decreased, although was not changed on mRNA expression. (C) We did not observed any significant changes of mRNA expression between disease groups compared to healthy subjects. *p <0.05; **p ≤0.01; ***p ≤0.001. [file Image_2.tiff]

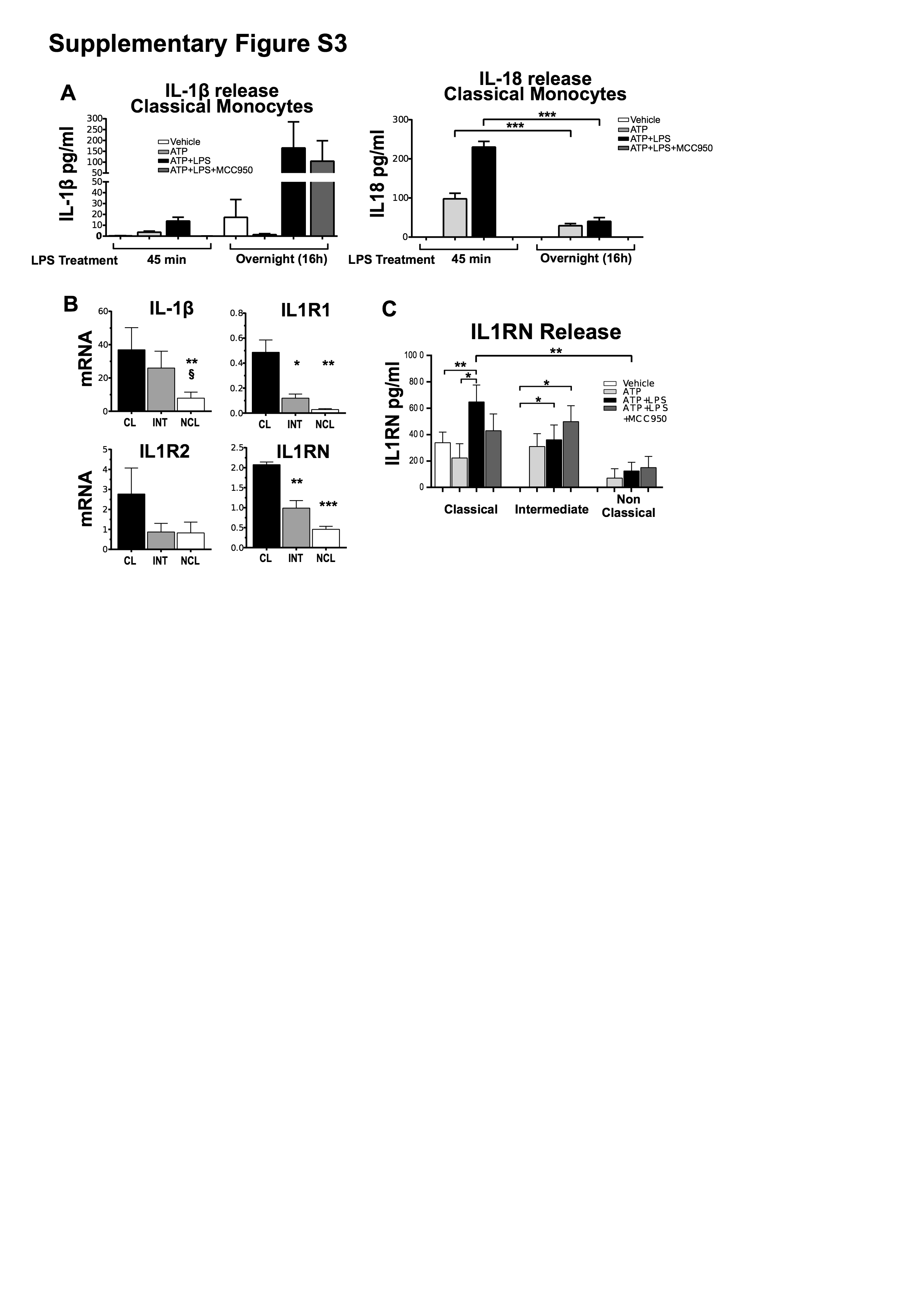

Supplement: Supplementary Figure 3 — The interleukin release profile shifts between canonical and rapid inflammasome activation. (A) A profile shift of inflammatory interleukins released from rapid (acute – 45min) to transcription-dependent inflammasome activation (overnight-16h priming with LPS and then next ATP activation) was observed in classical monocytes. Higher IL18 was secreted in response to rapid activation whereas IL-1β predominates upon canonical priming involving transcription of necessary protein components (n=3-6). (B) Expression of IL-1β-associated genes such as IL-1β, IL1R1 and IL1R2 as well as IL1RN varies significantly within monocyte subpopulations, presenting a higher basal expression in classical (CL) versus non-classical (NCL) monocytes. IL-1β itself was significantly lower expressed in NCL monocytes, and very variable in the other two subpopulations (n=5-12). (C) Release of interleukin 1 receptor antagonist (IL1RN) is significantly increased in monocyte subpopulations to a different extent upon dual inflammasome activation.Classical monocytes show a significant higher release of IL1RN upon inflammasome activation than non-classical monocytes. The release was not inhibited by MCC950 incubation (n=3-13). * vs classical; § vs intermediate;*p <0.05; **p ≤0.01; ***p ≤0.001. [file Image_3.tif]

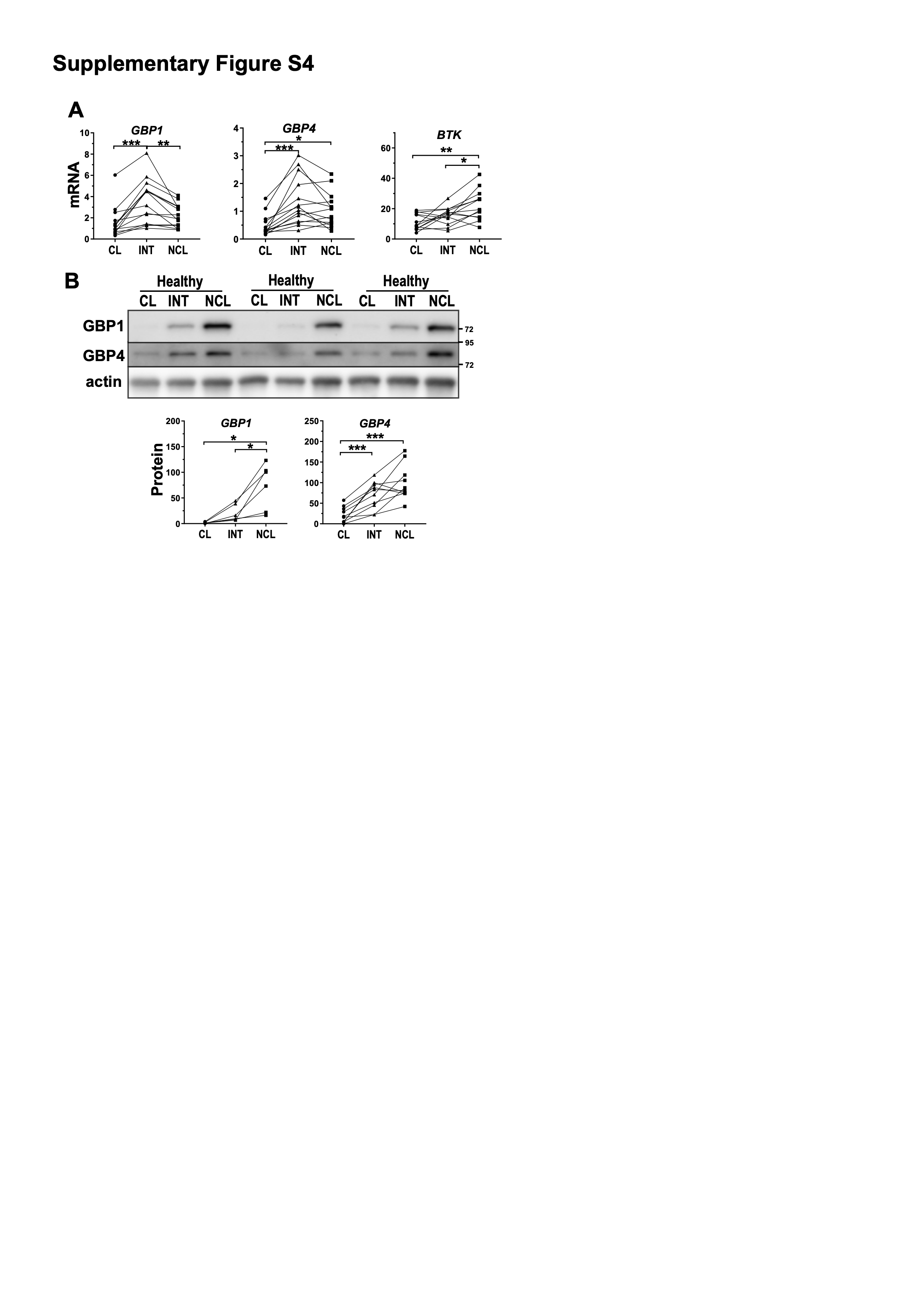

Supplement: Supplementary FIgure 4 — Basal expression analysis of other NLRP3 regulatory proteins. (A) mRNA expression of GBP1 was significantly higher expressed in intermediate monocytes and GBP4 as well as BTK show a significant increased in non-classical monocytes. (B) The protein levels of both GBP1 and GBP4 displayed an increase in intermediate and in non-classical monocytes as we showed for the other members of the family. *, p <0.05; ** p ≤0.01; *** p ≤0.001. [file Image_4.tiff]
